# The Role of Dietary Education in Cardiac Rehabilitation

**DOI:** 10.3390/nu17061082

**Published:** 2025-03-19

**Authors:** Joanna Popiolek-Kalisz, Michal Mazur, Francesco Perone

**Affiliations:** 1 Department of Clinical Dietetics, Medical University of Lublin, ul. Chodzki 7, 20-093 Lublin, Poland; joanna.popiolek-kalisz@umlub.pl (J.P.-K.);; 2 Department of Cardiology, Cardinal Wyszynski Hospital in Lublin, al. Krasnicka 100, 20-718 Lublin, Poland; 3 Cardiac Rehabilitation Unit, Rehabilitation Clinic ‘Villa delle Magnolie’, 81020 Castel Morrone, Caserta, Italy; francescoperone1988@gmail.com

**Keywords:** cardiac rehabilitation, myocardial infarction, nutritional education, secondary prevention, dietary education, personalized diet

## Abstract

Cardiovascular disease remains a leading cause of death globally; however, most cases could be prevented by addressing modifiable risk factors, such as unhealthy lifestyle factors, including diet. These aspects are also crucial in secondary prevention. Cardiac rehabilitation programs are vital in improving cardiovascular outcomes, and apart from recommended pharmacotherapy, they focus on lifestyle modifications, including exercise, a healthy diet, and smoking cessation. The aim of this review was to summarize the evidence on the role of dietary education in cardiac rehabilitation programs. The available data show that nutritional recommendations play an important role in cardiac rehabilitation programs, with the Mediterranean diet being widely recommended for its cardiovascular benefits. Adherence to dietary recommendations in the course of cardiac rehabilitation has been linked to improved metabolic and cardiovascular outcomes; however, further studies with long-term follow-up are needed. Moreover, while challenges in following dietary recommendations exist, individualized care and support are essential for successful outcomes in cardiac rehabilitation programs. Including dietary education is an important part of cardiac rehabilitation after myocardial infarction; however, more studies are needed to investigate the role of individualized dietary support and personalized education in cardiac rehabilitation.

## 1. Introduction

Cardiovascular disease (CVD) is the main cause of death in the European Union, accounting for up to 45% of all deaths [1]. It is estimated that addressing modifiable risk factors, including unhealthy habits, can prevent the majority of CVD cases and significantly improve the overall public health [2]. The registry by Nadarajah et al. [3] involved 3620 patients hospitalized at 287 centers in 59 countries due to non-ST segment elevation myocardial infarction, and collected data on patients’ characteristics, treatment outcomes, and guideline-compliant interventions, such pharmacotherapy, lifestyle modification advice, or referral to cardiac rehabilitation (CR). This registry aimed to identify barriers to the optimal management of this condition [3]. Myocardial infarction can be caused by different mechanisms; however, the most common one, myocardial infarction type 1, results from the rupture or erosion of an atherosclerotic plaque, which leads to acute closure of the coronary artery at the epicardial level [4,5].

On the other hand, malnutrition is a common problem in patients with coronary artery disease, which results in a poorer clinical prognosis. According to a study by Basta et al. [6], as many as 55% of patients with myocardial infarction had malnutrition. These individuals had an increased risk of death from various causes compared to that of patients with a normal nutritional status. Therefore, it is important to adequately assess a patient’s nutritional status after a myocardial infarction, as prompt diagnosis and treatment can help reduce the risk of complications, re-hospitalization, and death [6].

The introduction of CR is crucial for patients with CVD, as it is a comprehensive intervention that focuses on various aspects of health. The pillars of CR include pharmacological treatment of the underlying disease and comorbidities to control symptoms and improve cardiac function, along with lifestyle modifications, including a healthy diet, regular physical activity, and smoking cessation, which are key to improving heart health. Improving psychosocial well-being also plays an important role in the CR process, helping patients cope with the stress and emotions associated with the disease [7]. As already highlighted, CR focuses on lifestyle modification, which is sometimes limited to providing tailored exercise programs, while on the other hand, nutrition is an important lifestyle aspect related to CVD. The purpose this review is to present the available data on the role of dietary education and interventions in the course of CR after myocardial infarction.

The PubMed database was searched with the terms “cardiac rehabilitation” AND (“diet” OR “dietary education” OR “nutrition” OR “nutritional education”) without time or language limitations, and following PRISMA guidelines.

## 2. Assumptions of CR After Myocardial Infarction

Contemporary guidelines on CR, such as the European Association of Preventive Cardiology 2020 position statement and the British Association for Cardiac Prevention and Rehabilitation’s 2017 guidelines, emphasize the importance of providing quality care to patients after coronary events [8,9]. They also emphasize the importance of quality in the delivery of CR. An important element of these guidelines is the provision of comprehensive care and the development of a personalized treatment plan for each patient. The need for the appropriate monitoring of patients and regular assessments of progress in the rehabilitation process is also pointed out [8,9,10]. CR is not available worldwide, as only 40% of countries have programs of this kind [11]. Fewer than half of the patients in Europe who meet the criteria to receive CR opt for it, perhaps because of limited access or the low quality of services provided [12].

Selected countries offer comprehensive cardiac care programs for patients after myocardial infarction, including inpatient and outpatient treatment and CR. In Poland, the Coordinated Specialist Care after Myocardial Infarction (KOS-MI) program was fully funded by the National Health Fund. This ensured that patients received quality cardiac care after a myocardial infarction, which could improve their health and quality of life. A team of National Health Fund analysts summarized the results to date of the KOS-MI program, which consisted of providing patients with myocardial infarction treatment according to standards, CR, and specialized care. The program was implemented from October 2017 to December 2022, and benefited 68,000 patients. The KOS-MI program focused on comprehensive care for patients after myocardial infarction. The program was divided into four modules. Module I covered hospitalization, which included invasive treatment and diagnosis and care planning. Module II aimed at CR, which could be carried out in various ways 14 days after discharge from hospital. Module III covered electrotherapy and the implantation of special devices to improve patients’ health if applicable. Module IV focused on the long-term care of the patient for 12 months after myocardial infarction, providing support and health monitoring [13]. Units participating in the program were required to report on indicators of the quality of care and treatment results, which allowed for the continuous monitoring of the effectiveness of activities and the introduction of possible improvements [13]. The introduction of the program resulted in 31% lower death rates a year after myocardial infarction [14] and a 38% relative reduction in the risk of death within three years after myocardial infarction [15].

What is more, the results of the analysis by Shwaab et al. showed that the process of comprehensive CR significantly improved low-density lipoprotein cholesterol (LDL-C) control in patients after myocardial infarction in Germany [16]. The percentage of patients achieving concentrations below 70 mg/dL or a reduction of at least 50% increased from only 2% at the beginning of the study to an impressive 42% after an average of 22 days of therapy. This is an important finding that confirms the effectiveness of comprehensive CR in improving the health of patients after myocardial infarction [16].

A study by Andersson et al. was conducted to assess the impact of a five-year CR program on hospital treatment and sickness absence among women with CVD [17]. The study population consisted of 130 women under 65 years of age, divided into an intervention group (69 women) and a control group (61 women). The intervention program included intensive lifestyle modifications that led to a significant reduction in emergency department visits and hospitalization days in the intervention group, while the results remained the same in the control group. There were no significant differences in sickness absences between the two groups. The program helped to reduce the burden on the healthcare system, but did not affect the number of sick leaves [17].

The inadequate implementation and unstable quality of CR in coronary heart disease is a global problem. A Japanese study by Ohtera et al. aimed to understand the participation in CR in patients with coronary heart disease [18]. Data of patients after coronary intervention from 2017 to 2018 were analyzed. Of 87,829 patients, 32% participated in a CR program, where coronary artery bypass grafting was associated with higher participation rates than percutaneous coronary interventions. Patients from the Kiusiu region were more likely to participate in CR. The majority of the participants (92%) received training in pharmacology and 67 received training in nutrition. The implementation of CR in Japan is inadequate and needs to be improved [18]. The summary of the abovementioned studies is presented in Table 1.

## 3. Nutritional Recommendations in Patients After Myocardial Infarction

The report published by the World Health Organization in 2019 showed that proper investment in nutrition has the potential to save 3.7 million lives by 2025 [19]. Improper eating habits are one of the main causes of non-communicable diseases around the globe [20]. According to the European Society of Cardiology guidelines for the diagnosis and treatment of chronic coronary syndromes and for cardiovascular prevention, a proper diet for coronary artery disease should include a variety of nutrients [21,22]. It is recommended to consume at least 200 g of fruit and vegetables every day, as this is an important requirement to provide essential vitamins and minerals [23]. One’s diet should also contain between 30 and 45 g of fiber, coming mainly from whole grains [24]. Polysaccharides, including fiber, exert multifaceted effects on the CVD risk [25]. The recommended intake of nuts is 30 g per day and they must be unsalted [26]. In terms of fat sources, it is recommended to consume 1–2 portions of fish and at least 1 portion of fatty fish per week [27]. The consumption of red meat should be limited [28]. Saturated fats should make up less than 10% of the total energy value of the diet and should be replaced by unsaturated fats [29]. In terms of diet, care should be taken to keep the intake of trans fats as low as possible, i.e., less than 1% of the total energy value, which means avoiding processed products [30]. In addition, the salt intake should be limited to lower than 5 g per day, which is important for maintaining normal blood pressure [31,32]. As for alcohol, it is recommended to limit its consumption to 100 g per week and less than 15 g per day [33,34].

It is important that patients who are overweight or obese are aware of the benefits associated with weight loss and have clear goals to achieve. Appropriate interventions, such as nutrition programs, exercise, and behavioral support, are key to achieving meaningful results in the weight loss process. Recommended interventions include reducing the total energy intake, aiming for an energy deficit in the range of 500–750 kcal per day. This is particularly important when excessive body mass is accompanied by an increased waist circumference. The goal may require a loss of even more than 10% of the initial body mass in some patients. The recommended rate of weight loss is about 0.5–1 kg per week for at least six months [8]. A study by Borowicz-Bienkowska et al. analyzed the effect of brief CR, enriched with dietary counseling, on dietary habits in 44 patients following acute coronary syndrome [35]. The results were compared with a control group of 18 patients who did not participate in CR. After three months, a significant reduction in the caloric and cholesterol intake was observed in the study group. At the same time, there was observed an increase in the body mass index, which was, however, less pronounced than in the control group. The results indicate that CR may have a beneficial effect on improving dietary habits in patients after acute coronary syndromes [35]. The study by Luisi et al. showed an evaluation of the effectiveness of an educational program to improve the nutrition of patients with coronary artery disease [36]. The 160 patients were divided into two groups and 133 patients were included in the analysis. The intervention group participated in educational seminars and one-to-one sessions with a dietician. The results suggested that calorie intake, body weight, and body mass index were significantly reduced in this group compared to those in the control group, which may contribute to lower CVD risk [36].

In the context of this nutritional therapy, the Mediterranean diet stands out as one of the most well-known and well-studied dietary patterns, known for its potential benefits on cardiovascular health [34]. This diet promotes a balanced and varied approach to nutrition, focusing on eating natural and unprocessed foods, a moderate fat intake with olive oil as the main source, plenty of vegetables, limiting dairy, a reasonable consumption of fish, poultry, and wine, and limiting red meat, with daily fruit consumption [37]. The traditional Mediterranean diet is a plant-based way of eating that is rich in seasonal fruit and vegetables, legumes, and nuts, with extra-virgin olive oil considered the main source of fat. Whole grains are also consumed, usually in the form of sourdough bread. Fish is eaten moderately, usually 2–3 times a week. Fermented dairy, mainly in the form of yogurt and feta, appears in the diet most days. Red and processed meat is eaten infrequently, while preference is given to white meat such as chicken and game. Fresh and dried herbs and spices are used, and fresh lemon juice is used to season salads and dishes. Water is the main drink and wine is consumed in moderation, always during meals [38]. According to studies, adherence to the Mediterranean diet is associated with a 30% reduction in cardiovascular events and a 40% decrease in diabetes [39].

The Mediterranean diet is characterized by a high intake of olive oil, which is rich in oleic acid, and a low intake of saturated fatty acids [40]. Replacing carbohydrates with monounsaturated fats and proteins can also help lower blood pressure, improve lipid levels, and reduce the risk of CVD [41]. The health benefits of the Mediterranean diet have been recognized for more than fifty years, starting with the Seven Countries study, which revealed that the lower risk of death from heart disease in Greeks is linked to their diets rich in high-quality foods [42]. A recent analysis of 495 meta-analyses of randomized clinical trials examined the effects of different diet plans, including low-carbohydrate, high-protein, paleo, and Mediterranean diets, on anthropometric data and cardiometabolic risks. The results suggested that the Mediterranean diet had the most convincing evidence for positive health effects in the context of chronic conditions [43]. The impact of selected food products typical of the Mediterranean diet on the CVD risk is presented in Table 2.

In addition to the Mediterranean diet, the Dietary Approaches to Stop Hypertension (DASH) pattern is another widely recommended diet for patients with CVD, particularly for those with hypertension [31,50]. Moreover, the DASH pattern is also advised for obesity management [51], which is one of the main CVD risk factors that need addressing in the course of lifestyle management. Regarding the direct relationship with CVD, the study by Henzel et al. showed that lifestyle interventions that involved the DASH diet led to coronary plaque modification [52]. Moreover, DASH diet adherence was also reported as potentially useful in heart failure risk reduction [53]. DASH is a dietary pattern similar to the Mediterranean diet; however, it is more structured and focused on the limitation of salt intake [31] and the limitation of alcohol intake [41], with the constant acknowledgement of other Mediterranean diet principles. These modifications can lead to additional benefits for CVD management in CR. The DASH diet, similar to the Mediterranean diet pattern, supports a high intake of vegetables and fruits that are rich in fiber, vitamins, and antioxidative agents, which can reduce the risk of hypertension and also other aspects of metabolic syndrome [54,55,56]. Moreover, it emphasizes role of wholegrain products, and limits processed meat and saturated fat intake.

Fat intake modifications are another area of interest for CVD patients. In case of hypercholesterolemia presence, the saturated fat intake should be further reduced to below 7% [57]. However, the extreme limitation of fat consumption can potentially lead to an inadequate intake of fat-soluble vitamins and essential fatty acids, which can result in high-density lipoprotein cholesterol level reduction [58]. It has also been reported that low-fat diet adherence is low in the CR environment [59]. On the other hand, high-fat low-carbohydrate diet patterns, including the ketogenic diet, have gained a lot of attention over recent years [60]. However, the European Society of Cardiology guidelines indicate that fat intakes over 35–40% of one’s calories are associated with increased intakes of saturated fat and energy, which is why these dietary patterns need further investigation, particularly in terms of their long-term outcomes [57].

The diet for patients with CVD and diabetes needs to address both problems, and, as mentioned above, the Mediterranean diet is still the main advised dietary pattern for this group [61]. Glycemic control is an important aspect of metabolic balance; thus, the carbohydrate intake should be based on complex carbohydrates with a limited sugar intake, particularly from sweetened drinks [62]. Moreover, increased protein intake can bring additional benefits [63], but a shift from animal-based to plant-based patterns is still recommended [64].

To conclude, the regular monitoring of progress and adjusting diet plans as necessary is one of the key elements of effective behavioral nutrition interventions. It is also important to promote regular physical activity, which is an excellent complement to a healthy diet. Helping patients to make lasting changes to their eating habits should include motivation, education, and emotional support. Even after treatment, care and support are essential to help patients maintain healthy habits in the long term. Central to this is the team’s holistic approach, which takes into account both the physical and mental aspects of the patient, contributing to the success of dietary changes [65].

## 4. Dietetics in CR After Myocardial Infarction

Dietary recommendations with the participation of a nutritionist are considered an important part of CR quality assessment [66]. Patients in CR programs who suffer from other non-communicable diseases such as diabetes, hypertension, and lipid disorders should undergo a personalized evaluation and a thorough analysis of their health status. With personalized intervention in the area of dietary advice, they can maintain positive health outcomes. Working with a dietitian provides them with a better understanding of the relationship between their health and proper nutrition, and teaches them how to adjust their diet accordingly to improve their health [8]. An improved quality of life, control of the risk of CVD, and increased chances of living longer are all possible with an appropriate nutritional plan during therapy [67]. Dietary interventions in CR focus on modifying menus according to a patient’s personal cultural preferences, as well as training and counseling that take into account their demographic and psychological characteristics. It is important to develop educational materials tailored to patients’ educational level and health literacy, and to offer acceptable alternatives to harmful dietary choices [68]. Ethnic populations face a dilemma regarding risks and treatments—their risk of mortality and cardiovascular morbidity is higher, but, at the same time, they are less likely to participate in programs aimed at reducing these risks, such as CR [69,70]. South Asians in white-majority countries had higher rates of heart attacks, double the prevalence of diabetes, and higher abdominal obesity [71]. African-Americans experienced twice the rate of cardiac arrest and a 43% prevalence of hypertension compared to that of white participants [70]. Sociodemographic aspects are another important factor. Seventeen studies examined how age influences adherence to medical advice. In six of these, a significant relationship between age and the patients’ adherence to these indications was proven [72,73]. Although the relationship between gender and the adherence to doctors’ recommendations was not consistent across studies, an analysis of 14 publications showed that 6 of them noted a significant effect, suggesting that gender matters in this regard. In contrast, three studies showed that women were less likely to adhere to CR treatment recommendations compared to men [74,75]. One study revealed that there is a relationship between gender and age. The analysis suggests that older women are more likely to adhere to response control (CR) rules compared to younger women. This suggests that age may interact with the way women approach adherence to these rules, which may be important in the context of studying health behavior across age groups [76]. In contrast, nine studies investigated various aspects of work. Two of these studies indicated that white-collar employment promoted adherence to liability-related policies, whereas general work activity was associated with lower adherence to medical advice. In addition, one high-quality study revealed that higher levels of socioeconomic deprivation were associated with poorer adherence to advice [77]. Dietary recommendations in Brazil include 40% of CVD programs, indicating the importance of adequate nutrition for cardiovascular health in the country [78].

In British CR, nutrition education focuses on promoting a healthy dietary pattern as a whole, rather than focusing on individual nutrients. The results from a large cohort study (n > 120,000) indicate that altering the macronutrient intake (by adjusting the quantity or quality of food) has an impact on the CVD risk. Replacing a 5% intake of refined starches or added sugars with whole grains or a 5% intake of saturated fatty acids with unsaturated fatty acids reduced the risk of CVD in middle-aged and older adults [29].

### 4.1. Nutritional Effects of CR

The study by Froger-Bompas et al. aimed to assess the sustained positive impact of a CR program on dietary adherence among patients with coronary artery disease [79]. The authors analyzed data on the eating behavior of patients who participated in a CR program. The results of the study showed that participation in the program had a significant effect on increasing the participants’ adherence to dietary recommendations. Patients who regularly participated in the CR program were more likely to follow dietary recommendations in the long term. The study confirmed the association between participation in CR and long-term improvement in dietary habits in patients with coronary artery disease. These findings suggest important health benefits of involvement in a CR program in terms of dietary improvement in people with coronary artery disease [79]. The aim of the study by Hag et al. was to analyze the impact of the structure and procedures of CR programs on the eating habits of patients after myocardial infarction [80]. An analysis of data from 73 Swedish rehabilitation units and 5248 patients revealed numerous factors that may predict healthy eating habits one year after myocardial infarction. The most important elements were the presence of a medical director in the unit, a supportive team atmosphere, the formal training of nurses in dietetics, the provision of information on risk factors, and the investment of time in interactions with patients. The more positive attributes found in a particular facility, the greater the improvement that went into the patients’ eating habits. The results of this study may contribute to better CR planning and effective resource allocation to maximize patient benefit [80]. A study by Novaković et al. showed that patients who adhered better to healthy lifestyle principles at the start of CR had more favorable results in terms of glucose, triglyceride, and high-density lipoprotein cholesterol (HDL-C) levels [81]. At the end of the CR, significant improvements were noted in the patients who had previously adhered to these principles to a limited extent. In contrast, no significant changes were observed in the group of patients who strictly adhered to the healthy habits. The results suggest that the promotion of a lifestyle in the spirit of the Mediterranean diet may contribute to better adherence to healthy principles, especially in less advanced patients. The study included 121 post-myocardial infarction patients, and the CR program lasted 12 weeks and included both exercise and dietary workshops. The participants completed the Medlife Index Questionnaire before and after the CR [81].

According to the study by Fard et al. [82], participation in a CR program, which consisted of 24 exercise sessions and consultations with a nutritionist and psychiatrist, led to a significant reduction in the LDL-C levels and an increase in HDL-C [82].

Moreover, a study by Ślązak et al. [83] aimed to evaluate changes in the body composition, including the phase angle, in patients after myocardial infarction who participated in the KOS-MI CR program. The results of the study showed that patients after myocardial infarction who underwent the KOS-MI CR program displayed favorable changes in body composition, i.e., a reduction in visceral fat levels and levels of adipose tissue in the lower and upper limbs; however, this was without significant changes in the phase angle. The study suggested that the KOS-MI CR program may have had a positive effect on the body composition and health parameters of patients after myocardial infarction [83]. The study by Holmes et al. [84], on the other hand, aimed to examine the impact of dietary services on improving patient outcomes, and to evaluate the effectiveness of the Meats, Eggs, Dairy, Fried foods, fat In baked goods, Convenience foods, Table fats, Snacks (MEDFICTS) diagnostic test in a CR program. They investigated whether the use of dietary counseling and dietary assessment using the MEDFICTS test contributes to improved patient health outcomes. The results of the study showed that dietary services are associated with improved outcomes for patients participating in a CR program. In addition, the MEDFICTS diagnostic test was found to be an appropriate measure for assessing diet effectiveness among patients. The conclusion of the study is that dietary services are important for improving health outcomes for patients in cardiac rehabilitation programs. The MEDFICTS test is a useful tool for evaluating patients’ diets during CR after cardiac procedures [84].

In contrast, a study by Plüss CE et al. [85] investigated the long-term effects of an extended CR program after myocardial infarction or bypass surgery. In a randomized trial involving 224 people who had recently undergone myocardial infarction or surgery, 111 participants underwent long-term CR and 113 were the control group. In addition to standard rehabilitation, the intervention group received additional stress management sessions and nutritional counseling. After 5 years of follow-up, 48% of the participants in the intervention group experienced a cardiac event compared to 60% of the participants in the control group (risk ratio 0.69). The number of non-fatal myocardial infarctions and hospitalizations also decreased in the rehabilitation group. These findings suggests that long-term CR reduces the long-term risk of cardiovascular events [85]. The aim of the study by Rasmussen et al. [86] was to assess differences in the feeding behavior and clinical outcomes in patients participating in traditional CR and intensive Pritikin rehabilitation. An analysis of cardiac registry data included patients from a single hospital between 2015 and 2021, comparing the traditional CR group (n = 420) with the Pritikin intensive CR group (n = 1005). The eating behavior was examined using the Rate Your Plate index and various clinical risk factors such as lipid levels, blood pressure, anthropometric measurements, and psychosocial well-being were assessed. The results showed that both rehabilitation programs provided significant benefits, but the participants in Pritikin’s intensive CR showed greater improvements in the eating behavior and clinical outcomes. The smoking status did not prove to be a significant predictor of the eating behavior or clinical outcomes in both groups. Overall, the patients participating in the intensive Pritikin rehabilitation achieved significantly better cardiometabolic health outcomes [86].

In contrast, the study by Duarte et al. [87] aimed to identify barriers of and facilitators to dietary recommendations for participants in a CR program in a low-resource context in Brazil. The study was conducted using a qualitative analysis that included group interviews with CR program participants. The barriers identified included a lack of access to healthy products, financial constraints, a lack of knowledge about healthy eating, and difficulty in changing eating habits. In contrast, the participants reported support from other people, motivation, access to information, and dietary guidance as facilitators [87]. In the study by Bertelsen et al. [88], 327 of 1364 patients were eligible for the program, 212 of whom chose to participate. The phase II CR was successfully completed by 192 participants (91% of the participants). The difference in adherence between the shared care CR group (53%) and the hospital-based CR group (54%) was small, with a relative risk of 0.98. The patients in the hospital-based CR group adhered better to the dietary and health education recommendations. Although 12% of the patients in the shared care CR group did not participate in the risk factor assessment, there was no significant difference in the risk factor improvement between the groups. Additionally, a quarter of the patients in both groups refused to participate in the physical training. These findings highlight the importance of individualized care and support to enable patients to fully participate in CR programs [88]. The importance of dietary recommendations in CR programs with the guidance of a nutritionist is highlighted as a crucial aspect of quality assessments. The summary of the abovementioned studies is presented in Table 3.

### 4.2. Dietary Education Providers

Maintaining healthy eating habits permanently can be difficult, as many people return to their previous habits after completing a CR program in as little as six months [59]. Making changes in one’s daily diet requires strong will and determination, as well as support from family and specialists. It is always important to discuss the nutrition plan with the patient, who should be involved in treatment decisions. The patient’s family and medical professionals should also be informed about the nutrition plan.

Nutrition education plays an important role in CR in the UK, but it is not clear how it is implemented. The study by James et al. [89] aimed to identify professionals involved in nutrition education in rehabilitation programs and determine the format and content of the classes. Fifty-four professionals participated in the study, with 49 centers educating primarily through nurses and dietitians. A proportion of 46.9% of the professionals were not qualified in nutrition. The education programs typically lasted eight weeks and included multiple nutrition sessions with a nutritional assessment at the start of the treatment and during rehabilitation. The main topic was the Mediterranean diet and other topics such as malnutrition were given lower priority. Professional qualifications, nutritional assessments, and screening for malnutrition should be improved [89].

It is important to raise awareness about the importance of appropriate nutritional changes to control cardiovascular risk factors as part of CR. If necessary, nutrition interventions should include behavior change models and adherence strategies. It is also possible to organize cooking classes for patients and their families during the rehabilitation program. In the case of comorbidities, patients’ individual needs should be taken into account and dietitians should collaborate with an interdisciplinary team [90]. To achieve CR goals, a multidisciplinary approach that includes exercise training, risk factor modification, psychological support, patient education, and nutritional recommendations is essential. With a comprehensive approach, it is possible to effectively reduce the risk of CVD and improve the patient’s overall health. The collaboration of various medical and health areas allows for a holistic approach to the secondary prevention and treatment of CVD, resulting in better therapeutic outcomes and improved quality of life of patients [91].

### 4.3. Psychological Aspects

According to the American Heart Association Exercise/American Association of Cardiovascular and Pulmonary Rehabilitation, one of the main goals of CR programs is to improve the emotional condition of patients [92]. Participation in CR after percutaneous coronary intervention contributes to reducing the burden of disease and the risk of cardiovascular events. A study by Douma et al. [93], involving 1682 patients, examined the impact of psychological factors on healthy habits after percutaneous coronary intervention. The results indicate a significant association between psychology and health behavior, and, in particular, the strong effect of optimism on dietary adherence. Participation in CR mitigates the negative effects of anxiety and pessimism, but may, at the same time, reinforce the association between stress and smoking habits. It was noted that patients with high levels of anxiety and pessimism may particularly benefit from participating in rehabilitation programs, and this should be taken into account in their design [93]. In contrast, a study by Gostoli et al. [94] analyzed the effect of CR on unhealthy behavior modification and the course of CVD, taking into account depression, anxiety, and psychosomatic disorders. The longitudinal study included 108 patients undergoing CR and 85 coronary artery disease patients who did not participate in the CR. The evaluation was conducted four times: at admission, at discharge, and 6 and 12 months after the start of the study. The results showed that the CR promoted physical activity and improved behaviors related to nutrition, stress management, and sleep quality, but had no significant effect on weight loss, the implementation of a healthy diet, or adherence to medical recommendations. They also noted that depression and psychosomatic disorders can significantly affect changes in health behavior. The authors emphasize the need for an integrated approach that incorporates assessments of psychiatric and psychosomatic factors in CR programs [94].

To conclude, the positive impact of CR programs on long-term dietary adherence and improvement in body composition was shown in various studies. On the other hand, the data focused on individualized nutritional counseling are limited. Moreover, barriers to following dietary recommendations, such as a lack of access to healthy products and financial constraints, exist in some contexts. Individualized care and support are essential to ensure patient engagement and optimal outcomes in CR programs. Overall, dietary recommendations play a significant role in improving cardiovascular health outcomes in patients participating in CR programs. To enhance the effectiveness of dietary education in CR, it is essential to provide individualized care and support tailored to each patient’s specific needs, which has already been proven to be effective in healthy individuals [95]. There are dedicated guidelines on methods of dietary counseling in CVD; however, a personalized approach could potentially be achieved through comprehensive nutritional assessments using tools typical for dietary care, e.g., 24 h food recalls, food frequency questionnaires, or dietary diaries. Moreover, using validated scores such as the 14-point Mediterranean diet score [96] could also help in the identification of specific areas for nutritional education. A personalized approach could better address individual dietary difficulties; however, incorporating group-based educational sessions, such as cooking classes, could also provide practical guidance while fostering peer support, which increases motivation and adherence [97]. Finally, regular follow-up consultations continued after CR programs could ensure continuous support and allow for adjustments to dietary recommendations, potentially improving long-term adherence and outcomes. These aspects including using validated tools and modern technologies including mobile applications need particular attention in future studies focused on CVD and CR.

## 5. Conclusions

The presented studies highlight the important role of diet-related interventions in CR after myocardial infarction. Personalized dietary counseling could improve metabolic outcomes and potentially reduce the risk of further incidents. The success of programs such as KOS-MI in Poland highlights the importance of holistic care that integrates pharmacotherapy, lifestyle changes, and psychosocial support. However, difficulties remain, such as the limited availability of and adherence to nutritional guidelines. To enhance the effectiveness of CR, a multidisciplinary approach that includes continuous monitoring and support, as well as personalized approach, is crucial. However, further research focused on long-term outcomes and mortality is needed to investigate the role of nutrition-focused interventions in CR.

## Figures and Tables

**Table 1:** The summary of the findings from studies focused on CR organization.

Study	Participants	Key Findings	Focus
Nadarajah et al. [3]	3620 patients, 287 centers, 59 countries	Identified barriers to optimal management of non-ST segment elevation myocardial infarction.	Patient characteristics and treatment outcomes.
Sobieszek et al. [13]	68,000 patients (2017–2022)	36% relative reduction in death risk, and improved access to and satisfaction with cardiac care.	Comprehensive cardiac care.
Shwaab et al. [16]	Patients after myocardial infarction	Significant improvement in LDL-C control, from 2% to 42%, achieving target levels post-therapy.	Lipid control in CR.
Andersson et al. [17]	130 women under 65	Reduced emergency visits and hospitalization in intervention group, no change in sickness absence.	Impact of lifestyle modifications.
Ohtera et al. [18]	87,829 patients in Japan	32% participation in CR, higher rates in certain regions, inadequate implementation noted.	Participation rates in CR.

**Table 2:** Selected food types and products and their effects on CVD risk.

Type of Food	Example Products	Role in CVD Risk Modulation
Fruit and vegetables	Tomatoes, citrus, spinach	Source of fiber, vitamins, and minerals; antioxidant and anti-inflammatory effects [23]
Olive oil	Extra-virgin olive oil	Rich in monounsaturated fatty acids; improves blood lipid profile [44]
Wholegrain products	Wholemeal bread, pasta	Source of fiber, vitamins, and minerals; beneficial effects on glucose and cholesterol levels [24]
Legumes	Chickpeas, lentils	High in protein and fiber; improve insulin sensitivity [45]
Nuts and seeds	Walnuts, almonds	Source of omega-3 fatty acids [46]
Fatty fish	Sardines, salmon	Source of omega-3 fatty acids; anti-inflammatory effects [47]
Herbs and spices	Oregano, basil	Anti-inflammatory and antioxidant effects; improve flavor of food without adding salt [48,49]

**Table 3:** Studies focused on the effects of dietary counseling in CR.

Study	Participants	Key Findings	Focus
Froger-Bompas et al. [79]	Patients with coronary artery disease	Increased adherence to dietary recommendations post-CR.	Dietary adherence.
Novaković et al. [81]	121 post-myocardial infarction patients	Improved glucose and lipid levels; better results in those with limited initial adherence.	Lifestyle adherence.
Fard et al. [82]	Participants in a CR program	Significant reduction in LDL-C and increase in HDL-C levels.	Lipid management.
Ślązak et al. [83]	Participants of KOS-MI program	Reduction in visceral fat levels and levels of adipose tissue in the lower and upper limbs.	Body composition.
Holmes et al. [84]	Patients in a CR program	Dietary services associated with improved outcomes in CR.	Effectiveness of the Meats, Eggs, Dairy, Fried foods, fat In baked goods, Convenience foods, Table fats, Snacks (MEDFICTS) diagnostic test.
Plüss et al. [85]	224 post-myocardial infarction patients	Long-term CR reduced cardiac events; additional counseling improved outcomes.	Long-term effects of CR.
Rasmussen et al. [86]	Traditional CR group (n = 420) and the Pritikin intensive CR group (n = 1005)	Patients participating in intensive Pritikin rehabilitation achieved significantly better cardiometabolic health outcomes.	Differences in feeding behavior and clinical outcomes.
Duarte et al. [87]	Participants in a CR program in Brazil	Identified barriers to dietary recommendations; support and motivation were facilitators.	Barriers to dietary adherence.
Bertelsen et al. [88]	Shared care CR and hospital-based CR groups	Patients in the hospital-based CR group adhered better to dietary and health education recommendations.	Role of individualized CR care in recommendation adherence.
Borowicz-Bienkowska et al. [35]	44 CR patients after acute coronary syndrome vs. 18 patients who did not participate in CR	Significant reduction in calorie and cholesterol intake in the intervention group.	Impact of the intervention on eating habits.

## Abbreviations

The following abbreviations are used in this manuscript:

CVD	Cardiovascular disease
CR	Cardiac rehabilitation
DASH	Dietary Approaches to Stop Hypertension
KOS-MI	Coordinated Specialist Care after Myocardial Infarction
LDL-C	Low-density lipoprotein cholesterol
